# Transplantation for Primary Sclerosing Cholangitis: Outcomes and Recurrence

**DOI:** 10.3390/jcm12103405

**Published:** 2023-05-11

**Authors:** Fuat H. Saner, Alexandra Frey, Björn-Ole Stüben, Dieter P. Hoyer, Katharina Willuweit, Martina Daniel, Jassin Rashidi-Alavieh, Jurgen W. Treckmann, Hartmut H. Schmidt

**Affiliations:** 1 Department of General- and Visceral- and Transplant Surgery, Essen University Medical Center, 45147 Essen, Germany; 2 Organ Transplant Center of Excellence, King Faisal Specialist Hospital and Research Center, Riyadh 11211, Saudi Arabia; 3 Department of Gastroenterology, Hepatology and Transplantation Medicine, Essen University Medical Center, 45147 Essen, Germany

**Keywords:** cholestatic liver disease, cirrhosis, liver transplantation, disease recurrence, immunosuppression

## Abstract

Primary sclerosing cholangitis (PSC) is characterized by inflammation of the whole bile duct system. Liver transplantation is only approved as a curative treatment when it comes to end-stage liver disease. The aim of our study was to assess morbidity, survival rates and PSC recurrence and the impact of donor characteristics in long-term follow-up. This was an IRB-approved retrospective study. A total of 82 patients were identified who were transplanted between January 2010 and December 2021 for PSC. Among these patients, 76 adult liver transplant PSC patients and their corresponding donors were analyzed. Three pediatric cases and three adult patients with a follow-up within <1 year were excluded from further analysis. Median (range) age was 47 years (18–70) with a median (range) lab-MELD of 16 (7–40). Median (range) ICU and hospital stays were 4.6 days (0–147) and 21 days (1–176), respectively. The majority of patients suffered from Crohn’s disease or ulcerative colitis as a concomitant comorbidity (65.8%). The ten-year survival rate was 74.6%. A significantly lower lab-MELD score was identified in patients surviving for > 10 years (15 vs. 22, *p* = 0.004). Most patients (65%) passed in the first year following transplantation, with primary non-function (PNF), sepsis and arterial thrombosis being the most common causes of death. Donor characteristics did not affect patient survival. Patients with PSC show excellent 10-year survival rates. While the lab-MELD score significantly affected long term outcomes, donor characteristics did not affect survival rates.

## 1. Introduction

Primary sclerosing cholangitis (PSC) is an inflammatory disease which affects the bile duct system, while its etiology remains unknown [1]. The characteristic lesions are multifocal strictures of the intra- and/or extrahepatic bile ducts, which produce the typical cholangiographic picture. The course of the disease is not uniform but may lead to the development of cirrhosis and chronic liver failure [2]. Most patients with associated inflammatory bowel disease (IBD) typically present rectal sparing and backwash ileitis [3].

PSC is a complex and rare disease. Due to the high incidence of cholangio- and colorectal carcinomas, PSC is to be regarded as a pre-malignant lesion which requires a consistent surveillance strategy [2,4].

The clinical course of PSC shows high interindividual variability. Both very aggressive courses with the need for liver transplantation and stable disease courses over decades are possible. Approximately 30% of patients have histologic progression to cirrhosis within 5 years [5]. Median survival to liver transplantation or death are reported within 13–21 years [2]. The leading causes of mortality are malignancies of the biliary tract or colon, accounting for about 40% of deaths in PSC patients, while end-stage liver failure accounts for 33% of deaths [2].

About 40% of PSC patients require a liver transplant during their lifetime. In Scandinavian countries, for example, up to 15% of all liver transplants are performed in PSC patients [1]. The model for end-stage liver disease (MELD) score, which is mainly used for liver allocation, does not adequately reflect the prognostic risk in PSC. Accordingly, in EUROTRANSPLANT it is possible to apply for a so-called “standard exception” for PSC patients [6]. Additionally, in individual cases it is possible to apply for a “non-standard exception”, for example in the case of severe therapy-refractory pruritus. Patients transplanted with PSC as an underlying disease show excellent long-term survival, with survival rates of 87% at 5 and 83% at 10 years having been previously reported [7]. However, the recurrence of PSC (rPSC) in the donor liver is observed in approximately 20–30% of patients. Although data concerning retransplantation rates are lacking, based on expert opinion the rate of retransplantation for PSC patients seems higher compared to other diseases.

Beside ESLD, there are different indications for liver transplantation (LT) in PSC, such as recurring uncontrollable cholangitis, intractable pruritus and decompensated secondary biliary cirrhosis similar to primary biliary cholangitis (PBC) [8,9].

There are some reports indicating an rPSC rate of 20% within the first 5 years after transplantation [7,10]. Ulcerative colitis and younger age at transplantation have been reported as the main risk factors for recurrence [10]. The distinction between PSC recurrence and non-PSC-related post-transplant biliary strictures is important, which can also be present in up to 36% of transplanted organs for different reasons (e.g., post-transplant ischemia, infection, and chronic rejection) and negatively affects survival [7]. Moreover, there are two techniques available for bile duct reconstruction in liver transplantation, Roux-en-Y (RY) choledochojejunostomy or duct-to-duct anastomosis. In terms of long-term survival, rPSC and bile duct stricture, both techniques show similar results [11]. However, morbidity due to recurrent cholangitis is less common in patients with duct-to-duct reconstruction and therefore this method should be preferred whenever possible [12]. Data about the prognosis and causes of rPSC following liver transplantation are limited, which is why we evaluated our PSC patients.

## 2. Material and Methods

This retrospective, single-center, cohort study was approved by the local ethics committee (no: 22-10833-BO) and followed the Declaration of Helsinki. The ethics committee waived informed consent due to the retrospective design of this study. Organ procurement was standardized according to guidelines of the German Foundation of Organ Transplantation (DSO = Deutsche Stiftung für Organtransplantation). Recently, Heise et al. [13] summarized the current German guidelines.

**Figure 1:**
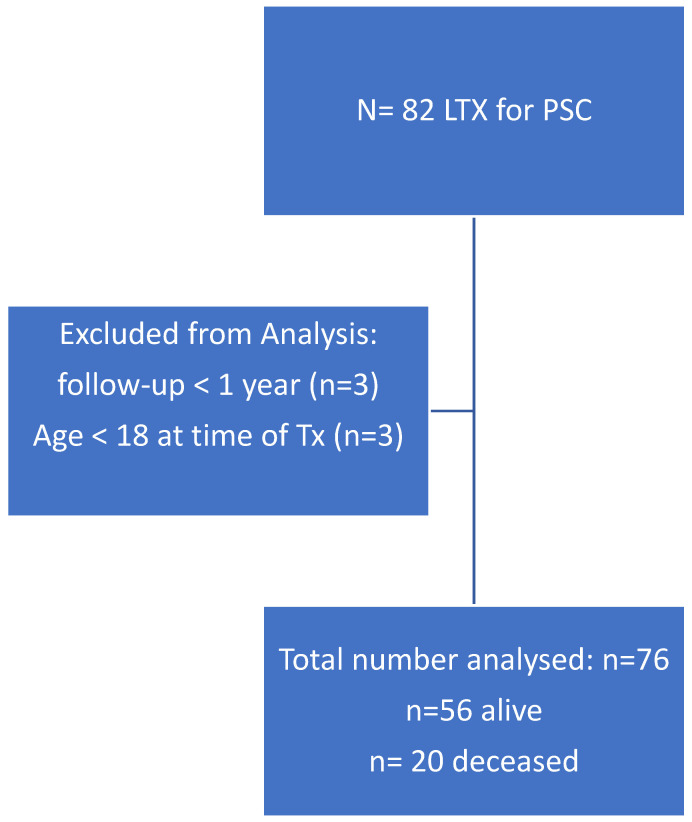
Between 1/2010 and 12/2021, eighty-two patients with PSC were transplanted. Among them, 76 patients were recruited to the study. PSC: primary sclerosing cholangitis; LTX: liver transplantation; Tx: transplantation.

From the chart review, we identified 82 patients who were transplanted between January 2010 and December 2021 with primary sclerosing cholangitis (PSC). Six patients were excluded from analysis, as three of them were pediatric transplants and three were adults with follow-ups of less than 1 year (Figure 1). We analyzed the data of 76 adult liver transplant PSC patients and their corresponding donors.

All liver transplantations were performed using standard surgical techniques (cava replaced technique, non-piggyback, first retrograde perfused via vena cava and no veno-venous bypass) and a standardized anesthesia protocol was applied to all patients. Patients were treated postoperatively in a single intensive care unit, applying standardized care consisting of triple immune suppression (corticosteroids, mycophenolate mofetile (MMF) and tacrolimus (TAC)). Daily trough levels of the calcineurin inhibitors (CNI) and TAC were measured for adjusting the daily dose (targeted TAC level 5–8 ng/mL). Corticoids were on a sliding scale and stopped after 4 weeks, and only patients with autoimmune hepatitis (AIH) were maintained on 5 mg prednisone. Most patients after discharge from hospital will be maintained on Everolimus and Tacrolimus.

Postoperative antibiotic and antifungal prophylaxes were not applied, since there were no signs of infection, regardless of whether the patient was a high-risk patient for fungal infection or not. As perioperative antibiotic prophylaxis in liver transplant patients, we used single-shot ampicillin/sulbactam (3 g). Regardless of the cytomegaly virus (CMV) status, all patients received CMV prophylaxis with Valganciclovir, adjusted to kidney function.

The diagnosis of rPSC was based on typical ERCP images and histologic findings in non-transplant PSC patients and exclusion of hepatic artery thrombosis.

### Statistical Analysis

Statistical analysis was performed with SPSS 28 statistical software (IBM SPSS Statistics; IBM Corporation, Chicago, IL, USA). Categorial data were reported as number (*n*) and percentage [%] and numeric data were presented as median and range. Normal distribution was assessed by Shapiro–Wilk test.

Statistical significance between groups was tested either with Student’s *t*-test, if the variable was normally distributed, or with Mann–Whitney-U, in the case of non-normal distribution.

To compare the results between groups, statistical significance was analyzed by χ^2^-test with Pearson approximation or Fisher’s exact test. A *p*-value of ≤0.05 was considered significant. Patient survival is shown with Kaplan–Meier curves.

## 3. Results

Median (range) age was 47 years (18–70) and median (range) lab-MELD was 16 (range, 7–40) at LT. Among the 76 patients, 63 (82.9%) were male.

Median (range) ventilation time was 15.5 (0–1255) hours and median (range) ICU stay was 4.6 (0–147.4) days. Median (range) hospital stay was 21 (1–176) days.

A total of 50 patients (65.8%) suffered from chronic inflammatory bowel disease. The majority of patients (43; 56.6%) had ulcerative colitis, while seven patients (9.2%) had Crohn’s disease.

### 3.1. Donor Characteristics

Median (range) donor age was 54 (5–79). Among the donors, the most common cause of death was hypoxia (*n* = 17; 22.4%), followed by subarachnoid hemorrhage (*n* = 16; 21.1%). The corresponding donor data for survivors and non-survivors were not different (Table 1B). A total of 16 patients (21.1%) were listed for retransplantation. Causes for retransplantation were PSC recurrence in six patients (7.9%) and bile duct complications, such as ischemia-type biliary lesions (ITBL), in four patients (5.3%). One patient developed primary non-function (PNF) and required retransplantation two days after transplantation.

#### 3.1.1. Bile Duct Reconstruction and PSC Recurrence

Biliary anastomosis by RY was performed in 30 cases (39.5%) and was used primarily between January 2010 and December 2014, while from January 2015 to March 2021 biliary reconstruction by duct-to-duct was performed in 46 cases (60.5%).

PSC recurrences following transplantation were encountered in six (7.9%) cases and occurred only in patients suffering from ulcerative colitis (*p* = 0.03). Among these six patients, in five patients bile duct reconstruction was performed by hepaticojejunostomy, and in one patient by duct-to-duct. rPSC recurrence was significantly more common in patients with hepaticojejunostomy than in patients with end-to-end bile duct reconstruction (*p* = 0.022). Median (range) time until listing for retransplantation due to PSC recurrence was 56 months (34–196).

All six patients were retransplanted. One patient is still alive, one patient passed from primary nonfunction and one patient developed bile leak, which could not be controlled, and passed from recurrent septic episodes. These two patients passed during their hospital stay. Three patients survived more than 3 years, two of them passed from chronic graft failure and one patient developed late hepatic artery thrombosis, followed by recurrent liver abscess, peritonitis and sepsis.

#### 3.1.2. Cytomegalovirus Infection

The cytomegalovirus (CMV) status was assessed in both donors (D) and recipients (R). In 34.2% of the cases, the donor and the recipient were positive for CMV (D+/R+). In total, 43.4% of patients were identified as intermediate risk (D+/R+ and D−/R+). Around one-third of patients (34.2%) presented a high-risk profile (D+/R−). The CMV D/R profile did not affect PCS recurrence or survival. CMV infection occurred in three patients within three months post-LT.

#### 3.1.3. Overall Survival Rate of the Total Cohort

**Figure 2:**
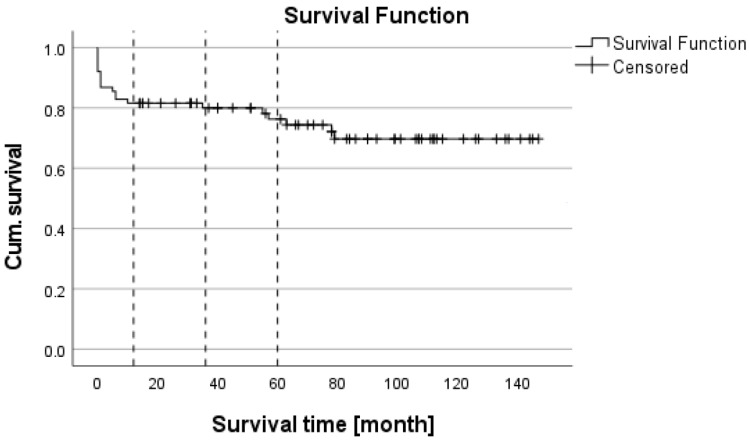
Kaplan–Meier survival analysis.

**Figure 3:**
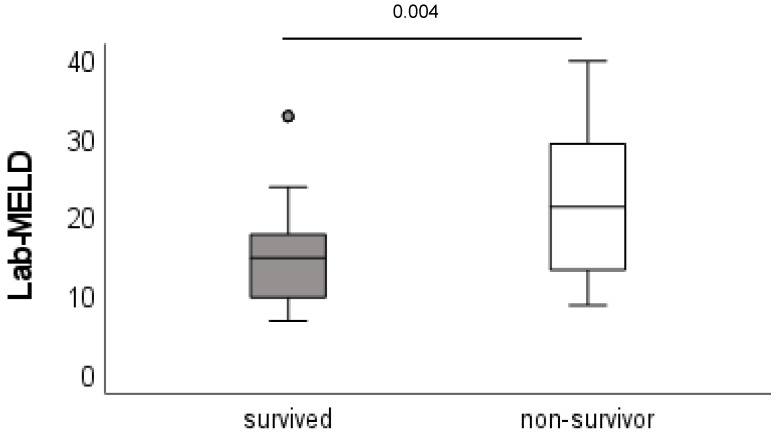
MELD score: survivors vs. non-survivors. MELD: model for end-stage liver disease, LT: liver transplantation.

Minimum follow-up time was 1 year. A total of 20 patients died within the follow-up period, 13 within the first year. The 1-, 3- and 5-year survival rates were 82.9%, 81.6% and 73.7%, respectively (Figure 2). Patients who survived had a significantly lower lab-MELD (model for end-stage liver disease) score (15 vs. 22, *p* = 0.004) and shorter ventilation time (12 h vs. 36 h, *p* = 0.008) compared with non-surviving patients. The occurrence of PSC recurrence was associated with a higher mortality (83%) compared to the group without recurrence (21%, *p* < 0.004). All other patient characteristics, such as age, body mass index (BMI) and preoperative liver-disease-associated complications, did not differ between groups (Table 1A and Figure 3).

Among the deceased patients, 45% died in hospital. The majority of patients died within the first year after transplantation (65% of all non-survivors). The main causes of mortality were primary nonfunction, sepsis and hepatic artery thrombosis (each: *n* = 3; 27.3%) (Table 2).

The ten-year survival rate was 74.6%. Within ten years, 20 patients (26.3%) died. The main cause of death was sepsis/septic shock (25%), followed by primary non-function (PNF) (*n* = 4, 15%) and hepatic artery thrombosis with graft loss (*n* = 4, 15%). While sepsis as the cause of death occurred in four patients within the first year after transplantation, one patient developed sepsis and septic shock after four years. PNF occurred immediately after transplantation.

Among the deceased patients, two patients died from a de novo malignant tumor (2.6%).

Among the relisted patients, 16 suffered from graft failure, with six patients dying on the waiting list. In ten cases, a suitable organ was allocated and successfully retransplanted (13.1%). Among these patients, six patients survived for at least one year.

### 3.2. Multivariate Analysis for rPSC and Mortality

Relating to death and rPSC, a binary regression analysis was performed to figure out the associations between the dependent variables (death or rPSC) and different variables (see Table 3). For death, lab-MELD and the kind of bile duct reconstruction were found to be independent variables.

The same analysis was performed for rPSC; however, an independent variable could not be identified.

## 4. Discussion

The most important result of our study was that PSC recurrence significantly affecting the survival of patients following transplantation occurred mainly in patients suffering from ulcerative colitis. Moreover, the surgical technique of bile duct reconstruction had a major impact on PSC recurrence. Duct-to-duct seems to be protective against PSC recurrence.

The advantages of duct-to-duct reconstruction include a shorter operation time, lower infection rate, more physiologic enteric functions and easier endoscopic access to the biliary tract. Bile duct stricture remains a major concern. However, there is an ongoing debate as to which kind of bile duct reconstruction should be preferred.

The analysis of the European Liver Transplant registry (ELTR) involving 1549 LT for PSC identified a recurrence of PSC (rPSC) in 259 patients (16.7%) after a median follow-up of 60 months, and this had a negative impact on patient survival [14].

Recurrence rates in our study were 7.9% and lower than in the previously published ELTR study. This discrepancy in recurrence rates can plausibly be explained by the smaller sample size in our study compared to the ELTR study (1549 patients vs. 76 patients). Another study evaluating ELTR data from 1980 to 2017 analyzed 6071 patients transplanted for PSC (representing, on average, 4% of all liver transplants). Re-LT caused by PSC recurrence (rPSC) was 8.6% in this cohort [15].

However, even studies with similar sample sizes than our study report conflicting results. Khettry et al. reported on 51 PSC patients with a follow-up of up to 14 years [16]; the rPSC rate was 11.8% following transplantation. Moncrief et al. [17] reported on 59 patients transplanted for PSC during a period of 17 years (1989–2006) and identified 25% of patients with rPSC. The differentiation of the recurrence of PSC from secondary bile duct changes independent of PSC after transplantation is difficult, which may explain the different results for PSC recurrence in different studies.

PSC recurrence patients were re-listed for transplantation in our study after a median time of 56 months. This is line with other studies giving the time for recurrence between 55 and 88 months [18].

The re-transplant rate in our study was 13.1%, which is in line with the study of Berenguer [15], who reported a retransplant rate of 16.1% (980/6071 patients). Visseren et al. [14] reported a retransplant rate for rPSC of 24.7%, and Ravikumar reported a retransplant rate for rPSC of 14.3%.

PSC recurrence in our study occurred only in patients suffering from ulcerative colitis. Additionally, in 83.3% of the cases, bile duct reconstruction was performed as a hepaticojejunostomy. In 2006, Gautam et al. [19] conducted a systematic review, but did not find a correlation between inflammatory bowel disease and rPSC. The limiting factor in this analysis was that only 5 out of 18 studies provided data about inflammatory bowel disease, which could be biased (364 of potentially 940 patients). Our study provided similar data to three previous studies. Cholongitas et al. [20] reported an rPSC rate of 13.5% and identified the presence of ulcerative colitis as an independent risk factor for rPSC. One year later, Alabraba et al. [21] reported on 230 patients undergoing liver transplantation for PSC; in this study, the rPSC rate was 23%.

This study identified colectomy before during LT to be protective against rPSC. Finally, the study of Hildebrand et al. [7] also demonstrated a close correlation between rPSC and ulcerative colitis. This close correlation between PSC and ulcerative colitis could be explained by the liver–gut link. PSC is a complex disease, with the etiology and pathogenesis based on multiple genetic and, to date, largely unknown environmental factors [1]. The results of genetic studies suggest an at least partial autoimmune genesis. There are associations with certain genetic polymorphisms of adaptive immunity, including the human leukocyte antigen (HLA) system and the regulation of T-lymphocytes [22]. Analogous to the pathogenesis of IBD, alterations in the gut microbiome, intestinal epithelial barrier disruption and abnormal immune responses to commensal bacteria may play a central role in the pathophysiology [23]. When ulcerative colitis occurs, the gut–liver interaction is disturbed. Liver-infiltrating lymphocytes promote inflammation, resulting in persistent inflammation and disease [24]. This could explain the close relationship between ulcerative colitis and rPSC. A meta-analysis from 2019 [18] identified colectomy before liver transplantation as having a protective effect against rPSC.

CMV infection is recognized as a risk factor for biliary complications, although the mechanism remains unknown [25,26]. Melrick et al. [27] supposed, in a pediatric patients cohort, that CMV infection prompts thrombosis in small arteries, which might explain the biliary complication. However, endothelial damage due to CMV infection was also reported [28]. In a previous study, Rashidie et al. showed that PSC was associated with a higher CMV infection rate compared to other diseases requiring liver transplantation [29]. However, our data do not support this finding.

Donor age was also discussed to increase the rate of rPSC. Hildebrand et al. [7] evaluated donor age in terms of rPSC and biliary stricture. The group showed that donor age was a predictor for rPSC (HR: 1.018; 95% CI: 1.006–1.030). Ravikumar et al. published in 2015 [10] a study which did not find a correlation between donor age and rPSC. One reason for the different results may be related to the sample size (Hildebrand *n* = 305 patients, Ravikumar *n* = 565 patients). Moreover, an epigenetic factor may also be considered. Hildebrand reported on the results from 10 German liver transplant centers for patients transplanted between 1990 and 2006, while Ravikumar reported the results of 6 liver transplant centers in the United Kingdom between 1990 and 2010. A group from Birmingham [21] identified ECD (extended criteria donors) as a significant risk factor. They speculated that ECD grafts may be exposed to a prolongated cold ischemia time with increased risk of immunogenic injuries caused by ischemia/reperfusion injury.

The results in our study regarding the correlation of donor age with rPSC confirm the results of Ravikumar. Steentraten [18] conducted a meta-analysis, including donor age as a confounder for rPSC. In this meta-analysis, 1310 patients were recruited and showed a pooled HR of 1.24 (95% CI: 1.07–1.45) per ten years.

Controversy exists regarding the type of biliary reconstruction, with hepaticojejunostomy (RY) or a duct-to-duct (DD) anastomosis. Until 2015, RY was the preferred technique in most centers, because studies had suggested reduced rates of biliary complications and improved graft and patient survival [30]. On the other hand, a duct-to-duct reconstruction provides a more physiological reconstruction of the biliary tract with preservation of the sphincter of Oddi activity and easier access to the bile duct postoperatively compared to RY [31,32]. Our study identified that rPSC occurred more commonly in patients with RY reconstruction. However, the number of six patients appears decidedly too low to formulate a general recommendation on how to perform biliary reconstruction. Wells et al. [11] evaluated seven retrospective studies comparing duct-to-duct vs. RY reconstruction. Among 692 patients who met the study criteria, the authors did not describe any difference regarding the outcomes, including 1-year survival, biliary strictures or rPSC. Similar findings are described in another meta-analysis from 2015 [12]. Pandanaboyana et al. [12] evaluated ten studies with 910 patients who fulfilled the eligibility criteria. They did not find any difference concerning biliary leakage rates, biliary strictures or rPSC. However, RY construction was associated with an increased rate of cholangitis, most likely due to more ascending infections.

In our study, the MELD score was the only factor which improved survival. Surviving patients had significantly lower MELD scores compared to non-survivors. Other studies reported similar results [10]. They found rPSC to be a main risk factor for mortality (HR 2.79, 95% CI = 1.87–4.16). However, ulcerative colitis did not affect the survival (HR = 0.95, 95% CI = 0.68–1.33). Another study reported similar long-term results but found different risk factors for mortality [7]. Hildebrand et al. [7] found, in 335 patients, bile duct stricture and rPSC with significant impacts on survival. The authors identified donor age, colitis, chronic rejection, bilirubin and INR at time of transplant as risk factors for biliary complications, and for rPSC they described donor age, colitis and INR at the time of transplant as risk factors. Both studies evaluated significantly more patients than our study. Our analysis did not support the results of these studies, and this could be related to the smaller sample size.

It is common sense that a higher MELD score is associated with an inferior post-transplant outcome. The MELD score was originally developed in 2000 to predict 3-month mortality in cirrhotic patients undergoing a TIPS procedure [33]. One year later, this scoring system was shown to be a reliable predictor for three-month mortality for cirrhotic patients [34]. Weismüller et al. [35] evaluated the liver transplantation outcome in German centers, after implementing the MELD score allocation in 2006. They found that MELD ≥ 30 was a strong predictor for postoperative mortality. Although the lab-MELD score in the non-survivor group was 22 in our study, it should be recognized that patients with PSC acquire their MELD score mainly with bilirubin, while INR and serum-creatinine are almost in normal ranges. Moreover, PSC patients with a MELD > 20 appear clinically more ill compared to other end-stage liver disease patients with alcohol-related or virus-related cirrhosis. To overcome this handicap, PSC patients receive exceptional MELD points if they meet specific criteria.

Our study has several limitations. As a retrospective study, it may have significant biases and the results may be less convincing than an RCT. The authors must rely on an accurate recording and cannot prove if the data are really reliable or not. The retrospective aspect may introduce selection bias and require a large sample size to identify differences in rare outcomes. However, our data concerning rPSC and survival correspond with other groups.

In conclusion, it seems that host factors are important in leading to rPSC in the new graft. There are still differences regarding different risk factors leading to rPSC described by different transplant groups. However, continuous updating, like the results of our study, may help to improve the knowledge about rPSC in patients following liver transplantation.

## Figures and Tables

**Table 1:** Patient characteristics and complications before LT (**A**) and donor characteristics (**B**). BMI: body mass index, LT: liver transplantation, MELD: model for end-stage liver disease.

**(A)**				
**Patient Characteristics**	**Total** ***n*** **[%]**	**Alive** ***n*** **[%]**	**Death** ***n*** **[%]**	***p*-Value**
Total patient number	76 [100]	56 [73.7]	20 [26.3]	-
Sex (male/female)	63 [82.9]/13 [17.1]	46 [82.1]/10 [17.9]	17 [85]/3 [15]	1.000
	median [range]	median [range]	median [range]	*p*-value
Age at LT [years]	47 [18–70]	46.5 [18–70]	47 [18–63]	0.540
Weight [kg]	72 [37–127]	74.5 [47–127]	65.5 [37–116]	0.186
BMI [kg/m^2^]	23 [15–41]	23 [15–47]	22 [19–31]	0.493
Lab-MELD	16 [7–40]	14 [7–33]	22 [9–40]	0.01
Warm ischemia time [min]	30 [0–51]	30 [19–44]	30 [0–51]	0.661
Hospital stay [days]	21 [1–176]	22 [12–82]	18 [1–176]	0.318
Ventilation time [h]	15.5 [0–1255]	12 [0–1255]	36 [0–769]	0.008
ICU stay [days]	4.6 [0–147.4]	4 [0–75]	5.4 [0.7–147.4]	0.126
Survival [month]	64 [--147]	81 [14–147]	3 [0–79]	0.001
Complication pre-LT	n [%]	n [%]	n [%]	*p*-value
Chronic intestinal diseases				
Crohn’s disease	7 [9.2]	4 [7.1]	3 [15]	0.371
Ulcerative colitis	43 [56.6]	29 [51.8]	14 [70]	0.195
Ascites	25 [32.9]	20 [35.7]	5 [25]	0.422
Splenomegaly	45 [59.2]	35 [62.5]	10 [50]	0.428
Esophageal varices	44 [57.9]	31 [55.4]	13 [65]	0.599
Hepatic encephalopaty	4 [5.3]	2 [3.6]	2 [10]	0.282
**(B)**				
**Donor Characteristics**	**Total** ***n* [%]**	**Alive** ***n* [%]**	**Death** ***n* [%]**	***p*-Value**
Total donor number	76 [100]	56 [73.7]	20 [26.3]	-
Sex (male/female)	44 [57.9]/31 [40.8]	34 [60.7]/21 [35.5]	10 [50]/10 [50]	0.430
	median [range]	median [range]	median [range]	*p*-value
Age at death [years]	54 [5–79]	53.5 [5–79]	59 [8–76]	0.360
Weight at death [kg]	80 [18–150]	80 [18–150]	76 [52–150]	0.421
BMI [kg/m^2^]	26 [15–46]	26 [15–46]	27 [22–44]	0.391
Cold ischemia time [h:min]	7:05 [0:0–14:00]	7:11 [0:0–12:26]	6:30 [4:00–14:00]	0.967
Donor Risk Index	1.7 [1.1–2.4]	1.6 [1.1–2.4]	1.8 [1.2–2.4]	0.123
Cause of death	n [%]	n [%]	n [%]	*p*-value
Hypoxia	17 [22.4]	12 [70.6]	5 [29.4]	0.371
Subarachnoid haemorrhage	16 [21.1]	12 [75]	4 [25]	
Cerebrovascular	13 [17.1]	9 [69.2]	4 [30.8]	
Intracerebral haemorrhage	13 [17.1]	10 [76.9]	3 [23.1]	
Trauma	5 [6.6]	2 [40]	3 [60]	
Living donation	2 [2.6]	2 [100]	-	
Others	7 [9.2]	7 [100]	-	

**Table 2:** Cause of death of LT patients.

Cause of Death	*n* [%]
Deceased	20 [26.3]
Deceased within first year after LT	14 [70]
Sepsis	4 [30.8]
Primary nonfunction	3 [23.1]
Hepatic artery thrombosis	3 [23.1]
Bile leak	2 [15.4]
Early graft dysfunction	1 [7.7]
Graft vs. host disease	1 [7.7]
Deceased between 1 and 3 years after LT	1 [5]
Cholangio-cellular carcinoma	1 [100]
Deceased >3 years after LT	5 [25]
Cholangio-cellular carcinoma	2 [40]
Chronical transplant failure	1 [20]
Sepsis	1 [20]
Late artery thrombosis, liver abscess and sepsis	1 [20]

**Table 3:** Multivariate analysis; death and rPSC are dependent variables.

	Multivariate Analysis	
	OR [95% CI]	*p*
Lab-MELD (continuous)	1.12 [1.04, 1.20]	0.002
Ulcerative colitis (presence)	1.97 [0.47, 8.37]	0.357
Crohn’s disease (presence)	3.59 [0.42, 30.74]	0.244
Donor Risk Index	2.42 [0.60, 9–87]	0.217
Cold ischemia time [h]	1.07 [0.80, 1.43]	0.645
Hepaticojejunostomy (presence)	5.57 [1.62, 19.15]	0.006

## Data Availability

The datasets generated and/or analyzed during the current study are not publicly available due to patients’ privacy.

## Abbreviations

BMI: body mass index; CMV: cytomegaly virus; ELTR: European Liver Transplant Registry; ESLD: end-stage liver disease; IBD: inflammatory bowel disease; ITBL: ischemia-type biliary lesions; LT: liver transplantation; MELD: model for end-stage liver disease; MMF: mycophenolate mofetil; PBC: primary biliary cholangitis; PNF: primary nonfunction; PSC: primary sclerosing cholangitis; rPSC: recurrence of primary sclerosing cholangitis; RY: Roux-en-Y; TAC: tacrolimus; Tx: transplantation
